# Comparative Analysis of Long-Term Outcomes in Valve-Sparing Aortic Root Reimplantation: Full Sternotomy versus Mini-Sternotomy Approach †

**DOI:** 10.3390/jcm13092692

**Published:** 2024-05-03

**Authors:** Jakub Staromłyński, Adam Kowalówka, Radosław Gocoł, Damian Hudziak, Małgorzata Żurawska, Wojciech Nowak, Michał Pasierski, Wojciech Sarnowski, Radosław Smoczyński, Maciej Bartczak, Jakub Brączkowski, Sabina Sadecka, Dominik Drobiński, Marek Deja, Piotr Szymański, Piotr Suwalski, Mariusz Kowalewski

**Affiliations:** 1 Department of Cardiac Surgery and Transplantology, National Medical Institute of the Ministry of Interior and Administration, Centre of Postgraduate Medical Education, 02-507 Warsaw, Poland; j.staromlynski@cskmswia.gov.pl (J.S.); wojciech.nowak@cskmswia.gov.pl (W.N.); m.pasierski@cskmswia.gov.pl (M.P.); w.sarnowski@cskmswia.gov.pl (W.S.); r.smoczynski@cskmswia.gov.pl (R.S.); m.bartczak@cskmswia.gov.pl (M.B.); j.braczkowski@cskmswia.gov.pl (J.B.); s.sadecka@cskmswia.gov.pl (S.S.); d.drobinski@cskmswia.gov.pl (D.D.); p.suwalski@cskmswia.gov.pl (P.S.); 2 Thoracic Research Centre, Collegium Medicum, Nicolaus Copernicus University, Innovative Medical Forum, 85-067 Bydgoszcz, Poland; akowalowka@sum.edu.pl; 3 Department of Cardiac Surgery, Upper-Silesian Heart Center, 40-635 Katowice, Poland; rgocol@sum.edu.pl (R.G.); dhudziak@sum.edu.pl (D.H.); mdeja@sum.edu.pl (M.D.); 4 Department of Cardiac Surgery, School of Medicine in Katowice, Medical University of Silesia, 40-055 Katowice, Poland; 5 Clinical Cardiology Department, National Medical Institute of the Ministry of Interior and Administration, 02-507 Warsaw, Poland; malgorzata.zurawska@cskmswia.gov.pl (M.Ż.); piotr.szymanski@cskmswia.gov.pl (P.S.); 6 Cardio-Thoracic Surgery Department, Heart and Vascular Centre, Maastricht University Medical Centre, 6229 HX Maastricht, The Netherlands; 7 Department for the Treatment and Study of Cardiothoracic Diseases and Cardiothoracic Transplantation, IRCCS-ISMETT, 90127 Palermo, Italy

**Keywords:** minimally invasive surgery, aortic root, mini-sternotomy, extracorporeal circulation, aortic valve disease

## Abstract

**Background:** Aortic valve-sparing aortic root replacement (VSARR) David procedure has not been routinely performed via minimally invasive access due to its complexity. **Methods:** We compared our results for mini-VSARR to sternotomy-VSARR from another excellence center. **Results:** Eighty-four patients, 62 in the sternotomy-VSARR group and 22 in the mini-VSARR group, were included. A baseline, the aneurysm dimensions were higher in the mini-VSARR group. Propensity matching resulted in 17 pairs with comparable characteristics. Aortic cross-clamp and cardiopulmonary bypass times were significantly longer in the mini-VSARR group, by 60 and 20 min, respectively (*p* < 0.001). In-hospital outcomes were comparable between the groups. Drainage volumes were numerically lower, and hospital length of stay was, on average, 3 days shorter (*p* < 0.001) in the mini-VSARR group. At a median follow-up of 5.5 years, there was no difference in mortality (*p* = 0.230). Survival at 1, 5 and 10 years was 100%, 100%, and 95% and 95%, 87% and 84% in the mini-VSARR and sternotomy-VSARR groups, respectively. No repeat interventions on the aortic valve were documented. Echocardiographic follow-up was complete in 91% with excellent durability of repair regardless of the approach: no cases of moderate/severe aortic regurgitation were reported in the mini-VSARR group. **Conclusions:** The favorable outcomes, reduced drainage, and shorter hospital stays associated with the mini-sternotomy approach underscore its potential advantages expanding beyond cosmetic outcome.

## 1. Introduction

Aortic root aneurysms pose a significant clinical challenge, often necessitating surgical intervention to mitigate the risk of catastrophic events [1,2]. While the conventional full sternotomy approach has long been the preferred method for aortic aneurysm repair, recent advancements have seen the emergence of valve-sparing techniques as endorsed by current guidelines, particularly when addressing aortic regurgitation resulting from aneurysmal widening of the aorta [3,4,5,6].

Minimally invasive approaches, encompassing minimal access and innovative cannulation techniques, present a promising avenue for aortic root aneurysm repair [7]. Despite initial challenges, studies have hinted at the potential for these techniques to yield outcomes at least as commendable as those achieved through standard approaches [8,9,10]. Furthermore, the broader benefits associated with the application of minimally invasive cardiac surgery techniques are anticipated in this specific patient cohort [11]. These advantages encompass improvements in quality of life, pain management, reduced blood loss, and shorter hospital lengths of stay. However, the existing body of data in support of these potential benefits remains insufficient [10], prompting the need for a comprehensive investigation into the comparative effectiveness of full sternotomy and minimally invasive approaches in valve-sparing aortic root replacement (VSARR) for aortic aneurysms. This study aims to bridge this knowledge gap by systematically evaluating the long-term outcomes of VSARR performed via full sternotomy and mini-sternotomy, shedding light on the optimal surgical strategy for this critical patient population.

## 2. Materials and Methods

### 2.1. MIRAGE Registry

The current study is a sub-analysis of the Minimally Invasive Aortic Root and Aorta surGery rEgistry (MIRAGE, NCT: 04814238). The study is registered, conforms to the provisions of the Declaration of Helsinki (as revised in 2013), and was approved by the local ethics committee (CSK MSWiA/KE/215/2018), and each patient signed an informed consent for treatment and the use of personal data. Between 2011 and 2022, 617 patients underwent aortic surgery at our institution. Exclusion criteria for the minimally invasive approach were the following: active aortic valve endocarditis, redo surgery, acute type A dissection, and concomitant cardiac procedures expanding beyond coronary artery bypass to the proximal right coronary artery. No age restrictions were imposed. A total of 249 consecutive elective patients (40.3%) were treated with a minimally invasive approach via an upper partial sternotomy; of those, after further exclusion of the minimally invasive Bentall procedure, supracoronary ascending aorta replacement, isolated sinus of Valsalva remodeling and other procedures, 22 (8.8%) patients remained who underwent isolated mini-VSARR. These were compared to isolated sternotomy cases performed within the same time frames in another excellence center (sternotomy-VSARR). Operative risk was evaluated according to the European System for Cardiac Operative Risk Evaluation II (EuroSCORE II) [12]. In the early experience (2011–2013) aortic dilatation >60 mm was an exclusion criterion; that was later lifted with the progression on the learning curve, and patients presenting with aortic aneurysms ≤70 mm were included in the study as well. Details on patients’ inclusion have been previously published [11]. In brief, each patient underwent preoperative angio-computed tomography and echocardiographic examination to determine exact aortic position and dimensions. The presence of extensive aorta calcifications was not an exclusion criterion, provided the plaques were not located at the cannulation site; coronary angiography was performed in patients >40 years old; any deviations from the planned procedure were left to the discretion of the involved surgeon.

### 2.2. Surgical Technique

General anesthesia was administered following standard procedures. External defibrillator pads were affixed, and a 3D transesophageal echocardiography (TEE) probe was inserted for each patient. Additionally, INVOS^®^ cerebral oxygenation monitoring probes (Somanetics Corporation, Troy, MI, USA) were positioned. Detailed descriptions of the surgical technique can be found elsewhere [7]. A V-shaped partial upper sternotomy was performed, starting from the sternal notch and extending to the 3rd or 4th intercostal space, to expose the ascending aorta and aortic root. Following identification and mobilization of the innominate vein, the pericardium was opened, and 7–8 pericardial stay sutures were placed. Subsequent to systemic heparinization, direct aortic and right atrium appendage cannulation were carried out, with the EOPA arterial cannula (Medtronic, Inc., Minneapolis, MN, USA) positioned in the proximal portion of the aortic arch. Venous drainage was facilitated using a three-stage MC2X cannula (Medtronic, Inc.), inserted into the right atrium-inferior vena cava, and later pulled through a 1.5 cm sub-xiphoid incision with downward pressure on the right atrium. Cardiopulmonary bypass was initiated, with the utilization of a cell saver left to the discretion of the operating surgeon. Depending on the surgeon’s preference, the patient was gradually cooled down to 32–34 °C. Subsequently, left ventricular venting was achieved via the upper right pulmonary vein.

The aorta was subsequently cross-clamped and opened, with selective delivery of cold blood cardioplegia through both coronary ostia, administered at intervals of 20–25 min. While instances of retrograde cardioplegia delivery were not encountered, it should be noted that this approach allows for such a possibility. Surgical procedures for aneurysm excision, valve replacement, and aortic anastomoses followed the conventional sternotomy approach. Dacron grafts were utilized, and the decision to employ fibrin or Bioglue (CryoLife, Kennesaw, GE, USA) for hemostatic support at the aortic anastomosis sites was at the discretion of the operating surgeons. Bioglue was consistently applied to reinforce coronary button anastomoses during root procedures. Temporary pacing wires were inserted, and de-airing was facilitated through the Reverse Trendelenburg maneuver and active left ventricular filling before declamping the aorta and discontinuing cardiopulmonary bypass (CPB). Intraoperative transesophageal echocardiography (TEE) was subsequently performed to evaluate valve function, particularly for signs of insufficiency, in all patients. Cannulas were withdrawn, and protamine sulphate was administered at a 1:1 ratio to heparin, with additional doses required if the activated clotting time exceeded 140 s. Administration of blood products during the operation was determined by the anesthetist’s judgment. A single chest drain tube was inserted into the anterior mediastinum via subxiphoid access after removing venous cannulas. The pericardium was closed using interrupted sutures in the upper portion, and the sternum was approximated with steel wires. The choice between topical vancomycin paste application to sternal edges [13] or placement of gentamycin collagen sponge [14] between sternal halves was based on the surgeon’s preference. Sternotomy cases followed a standardized protocol.

### 2.3. Definitions and Follow-Up

Acute kidney injury (AKI) was defined according to Kidney Disease Improving Global Outcomes (KDIGO) criteria [15]. KDIGO criteria define AKI as a 0.3 mg/dL (≥26.5 mol/L) sCr increase in sCr from baseline within 48 h of surgery, a 50% sCr increase from baseline within 7 days of surgery, or a decrease in urine output below 0.5 mL/kg/h for 6 h. Residual aortic regurgitation (AR) was graded based on pressure half-time and classified in between ‘none’, ‘trace’, ‘mild’, ‘moderate’ and ‘severe’ according to Carpentier [16]. In the presence of bicuspid aortic valve (BAV), the Sievers type of BAV was recorded [17]. Follow-up visits were scheduled at 6, 12 and 24 months. Longer follow-ups were conducted telephonically. Survival data were obtained from the KROK registry [18].

### 2.4. Statistical Analysis

STATA MP v13.0 software (StataCorp, College Station, TX, USA) was used for all computations. Normally distributed continuous variables were expressed as mean ± standard deviation (SD) and as median and interquartile range (IQR). Nonparametric and parametric data were evaluated using either the Spearman rank-test or the Pearson test. The Kaplan–Meier curves were fitted and used for presentation of overall survival and compared mini-VSARR with sternotomy-VSARR using log-rank test where applicable. Propensity score matching was applied in order to balance possible confounding between the 2 study groups regarding selected variables in order to avoid any bias related to the initial selection of patients for mini-VSARR. The variables age, BMI, aortic aneurysm size, and comorbidities (diabetes, smoking, hypertension, CVD, hyperlipidemia, pulmonary hypertension, renal impairment) were included as matching parameters. Regression adjustment was then applied and resulted in improved precision for the continuous outcome, as described by Steyerberg [19]. A two-tailed *p* value of less than 0.05 was considered statistically significant for all statistical tests employed.

## 3. Results

### 3.1. Patient and Surgical Characteristics

The patient cohort consisted of 84 patients: 22 in the mini-VSARR group and 62 in the sternotomy-VSARR control group. Table 1 lists baseline characteristics; nearly 90% of patients in both groups were male. Patients in the sternotomy-VSARR group were significantly younger (39 (28–52) vs. 64 (49–65) (*p* < 0.001)), had lower BMI (25.1 (23.3–27.8) vs. 27.8 (25.9–30.7) *p* = 0.008) and had numerically fewer comorbidities. Aortic valve insufficiency was the primary indication for surgery in mini-VSARR in all 22 patients, while eight (12.9%) of the patients in the sternotomy VSARR had their AV intact. They, however, more often presented with a bileaflet aortic valve (43.5% vs. 4.5% (*p* = 0.001)).

**Figure 1:**
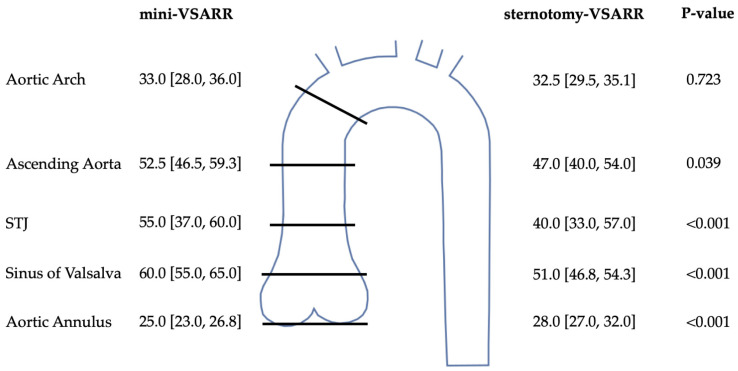
Aortic dimensions. Data are reported as median and interquartile ranges. VSARR, valve-sparing aortic root reimplantation; STJ, sinotubular junction.

Aortic dimensions are presented in Figure 1. Patients in the mini-VSARR group exhibited significantly wider aorta at the level of the sinus of Valsalva (60.00 (55.00–65.00) vs. 51.00 (46.75–54.25); *p* < 0.001); sinotubular junction (55.00 (37.00–60.00) vs. 40.00 (33.00–57.00); *p* < 0.001) and ascending aorta (52.50 (46.50–59.25) vs. 47.00 (40.00–54.00); *p* = 0.039). Aortic valve annuli were significantly smaller in mini-sternotomy cases (25.00 (23.00–26.75) vs. 28.00 (27.00–32.00); *p* < 0.001). There were no differences in aortic arch and descending aorta dimensions.

### 3.2. In-Hospital Course

All surgeries were elective. All were completed, and none required valve replacement. Seven patients (11.3%) in the sternotomy-VSARR group and three (13.6%) in the mini-VSARR group required reoperation for bleeding. There were two in-hospital deaths in the series, both in the sternotomy-VSARR group and both due to multiorgan failure in the ICU; three implantations of PPM and one stroke occurred in the sternotomy-VSARR group; in the mini-VSARR group, one PPM implantation was necessary. One conversion to sternotomy was performed after the completion of the procedure; the patient developed low cardiac output syndrome and required on-table central ECMO support, which was continued for 8 days; after cardiac rehabilitation, he was otherwise discharged uneventfully on post-op day 48.

To account for baseline differences in patients’ risk profiles, propensity score matching was performed and resulted in 17 pairs adjusted for age, BMI, aortic aneurysm size, and comorbidities. There were no differences in major postoperative complications (Table 2). The median aortic cross-clamp and cardiopulmonary bypass (CPB) time were significantly longer in the mini-VSARR group, by 60 and 20 min, respectively. We observed no differences in median ICU time, whereas the median hospital length of stay (HLoS) was, on average, 3 days shorter in the mini-VSARR group.

### 3.3. Follow-Up

**Figure 2:**
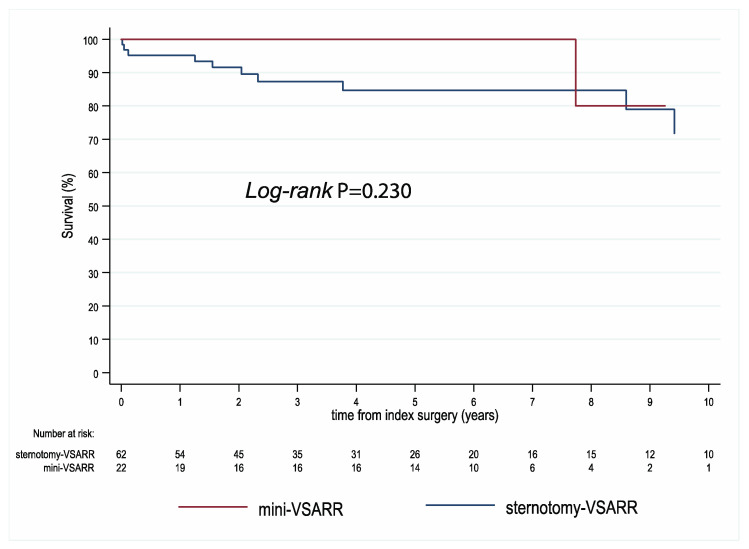
Kaplan–Meier survival curves for mini-VSARR and sternotomy-VSARR. VSARR, valve-sparing aortic root reimplantation.

Survival follow-up was 100% complete. The median follow-up regarding mortality was 5.5 years (IQR, 2.1–7.2) for mini-VSARR and 4.0 years (1.8–7.6) for sternotomy-VSARR. Kaplan–Meier curves for survival are presented in Figure 2, showing no observed differences in mid-term survival between the two techniques (*p* = 0.230).

No repeat interventions on the AV were documented during the follow-up period. In the sternotomy-VSARR group, one patient developed pericardial effusion and had pericardial drainage placement 2 months after discharge; another developed abdominal aorta dissection 1.5 years in the follow-up and underwent uneventful TEVAR.

**Figure 3:**
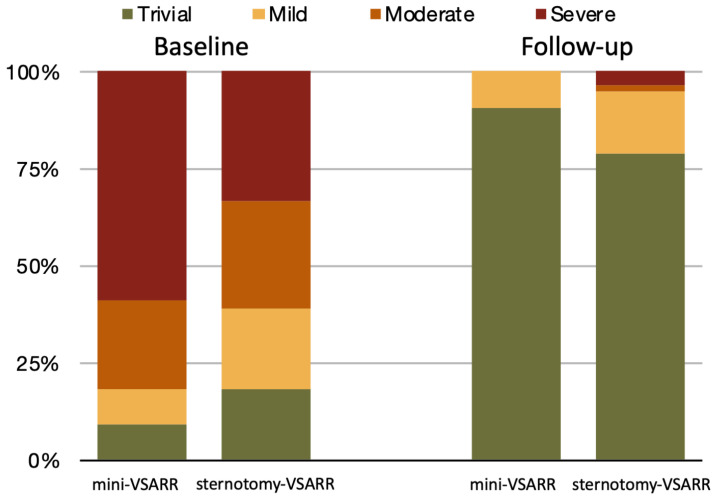
Echocardiography follow-up. VSARR, valve-sparing aortic root reimplantation.

Echocardiography follow-up was 91% complete for the mini-VSARR group and 81% complete for the sternotomy-VSARR group, with a median follow-up of 4.8 (1.1–6.6) years and 1.8 years (0.2–4.0), respectively. The durability of repair was excellent regardless of the approach: no cases of moderate/severe aortic regurgitation were reported in the mini-VSARR group, compared to three (5%) in the sternotomy-VSARR, as depicted in Figure 3. No signs of aorta widening at the level of aortic annulus were seen in either group. Further, no differences were noted in the reported NYHA score at the latest follow-up visit (1.26 vs. 1.20, *p* = 0.668).

## 4. Discussion

Cardiovascular and thoracic surgery continually strives for advancements that minimize surgical trauma, promoting faster recovery and improved patient outcomes [20]. In this study, we delve into the evolving landscape of aortic surgery, particularly focusing on the comparability and potential advantages of minimally invasive approaches. Our findings align with the broader literature, showcasing the adaptability and efficacy of these techniques in addressing complex aortic pathologies, specifically emphasizing valve-sparing aortic root reimplantation [3,4].

The current investigation resonates with previous works, such as the series by Shresta et al. [3,9], which underscores the safety and comparable outcomes of minimally invasive valve-sparing aortic root reimplantation in selected subjects. Our findings on the safety of the two approaches reflect those of Shresta et al., who recently updated their landmark database [9] and found 1-, 2-, 4-, and 6-year survival rates of 97, 97, 97, and 97% in the mini-access and 99, 96, 95, and 92% in the full sternotomy groups, respectively [21]. Reported CPB times and x-clamp times were 188.5 ± 35.4 and 126.2 ± 27.2 min, respectively, and were shorter than in our series: 226 (220–239) and 160 (158–171). The stepwise evolution from minimally invasive AVR to more intricate aortic root procedures, as emphasized by Shresta et al., is echoed in our findings. This gradual approach ensures a seamless transition and results comparable to conventional full sternotomy, reinforcing the feasibility and safety of such methodologies.

Expanding on this foundation, we draw insights from experiences reported by Mikus et al. [22] and Deschka et al. [23], who successfully extended the use of partial upper sternotomy for ascending aorta and aortic valve replacement. These studies not only demonstrate the versatility of the minimally invasive approach but also underscore its application in more complex aortic surgeries. Our study aligns with these experiences, affirming that the upper V-shaped mini-sternotomy provides a robust and adaptable platform for a spectrum of aortic surgeries, ranging from simple supracoronary replacements to intricate root procedures. Indeed, in the previous study [11], we were able to demonstrate excellent mid-term outcomes: within investigated follow-up (mean 3.1, max 7.7), survival was estimated at 95% without differences between procedures involving AVR or not: HR, 0.96; 95% CI: 0.26–3.59; *p* = 0.95. Remarkably, only one patient required reintervention within these time frames for acute valve thrombosis 24 months post-op. In the previous series, one patient underwent hemi-arch replacement via V-shaped mini-sternotomy. It has to be noted that such partial upper hemisternotomy has proved safe not only for aortic root surgery but also complex aortic arch procedures [24]. While acknowledging the importance of individual surgeon experience and patient factors, the study contributes to the growing body of evidence supporting the use of the minimally invasive technique in a broader range of aortic surgeries.

The current study’s aim was to compare mini-VSARR vs. sternotomy-VSARR; the procedural success and favorable early outcomes observed in our study further highlight the advantages of the mini-sternotomy approach. These benefits were confined to numerically lower drainage volumes and shorter hospital stays noted in patients undergoing minimally invasive procedures, underscoring potential benefits such as reduced blood loss and expedited postoperative recovery when compared to full sternotomy access. Importantly, no differences were seen between the two with respect to hard clinical outcomes, partially reflecting small sample size of the study groups but also excellent safety profiles of the two approaches.

Our findings echo the sentiment of other studies that advocate for the importance of preoperative imaging in patient selection for minimally invasive approaches. Both CT and echocardiography play integral roles in our patient selection process, aiding not only in the choice of optimal incisions but also in identifying critical factors, such as calcific plaques, that influence cannulation and anastomosis possibilities [25]. In selected cases, transitioning from mini-sternotomy to right mini-thoracotomy for root procedures is also feasible and safe [26,27,28]. Unfortunately, to date, not enough long-term data are available regarding the durability of such an approach.

It is crucial to acknowledge the risks and limitations inherent in adopting mini-sternotomy for patients undergoing valve-sparing root implantation. Risks include the potential for injury to adjacent structures, while limitations may stem from chest deformations or anatomical variations that complicate the procedure. Mini-sternotomy should be approached cautiously in redo surgeries or cases with significant valve insufficiency and minimal enlargement of the sinotubular junction, as the technical challenges in delivering cardioplegia may outweigh potential benefits. Furthermore, resistance from surgical teams, often due to concerns over increased initial surgical duration, may pose a hurdle to its introduction. Despite these challenges, meticulous pre-operative planning and a commitment to overcoming the learning curve are essential for successful implementation of mini-sternotomy in patients with aortic root aneurysm and valve pathology, ultimately offering a minimally invasive alternative with favorable outcomes.

Unlike the case with full sternotomy [29], mini-sternotomy has only recently entered the field of VSARR; thus indeed, the long-term results of such an approach are unique. Our study highlights the durability, freedom from reinterventions and symptom relief that are comparable to sternotomy. Operative times are longer, but in selected elective patients, this does not translate into longer ICU stays or increased propensity for complications. On the contrary, due to shorter ICU recovery and commencement of cardiac rehabilitation, HLoS durations were shorter in the mini-VSARR group. Cosmetic outcome is just an addition but should always be taken into account.

While acknowledging the successes, it is crucial to recognize the limitations inherent to a double-center, retrospective study design. This intentional design aimed to enable a comparative analysis of different surgical approaches across institutions. The authors believe that contrasting isolated mini-VSARR with isolated sternotomy-VSARR from another institution provided a more appropriate comparison than juxtaposing mini-VSARR against sternotomy VSARR combined with coronary artery bypass grafting (CABG) and/or mitral valve surgery performed within a single center. While acknowledging this deliberate choice, it is essential to recognize the inherent limitations associated with double-center studies. Moreover, although our propensity-matched results suggest the mini-VSARR procedure is not inferior to sternotomy-VSARR in terms of safety, the mini-VSARR cohort exhibited significantly longer CPB and aortic cross-clamp times. These are factors that could potentially impact outcomes, albeit not observed in our study due to its constrained size. Hence, larger-scale investigations are imperative to conclusively determine the equipoise in safety between the two approaches. Furthermore, while our study revealed a shorter HLoS in the mini-VSARR group, it is essential to acknowledge that this discrepancy may stem from center-specific discharge protocols rather than inherent differences in surgical techniques. Only through a prospective, randomized trial employing an algorithm-based discharge protocol can the superiority of the mini-VSARR approach in this aspect be definitively confirmed. Initially, the study was restricted to elective cases due to safety concerns, excluding patients with decompensated heart failure attributable to aortic regurgitation (AR) or those deemed higher risk. As our experience with mini-valve-sparing aortic root replacement (mini-VSARR) grows, we anticipate the inclusion of higher-risk patients, such as those with broader aneurysms or aortic arch involvement, or undergoing hybrid procedures involving percutaneous coronary intervention (PCI), thoracic endovascular aortic repair (TEVAR), or transcatheter mitral edge-to-edge repair. A notable limitation is the relatively small sample size of patients included in the study. We acknowledge this limitation, recognizing that minimally invasive surgery for aortic disease, particularly mini-VSARR, is infrequently performed. However, it is worth noting that our experience represents one of the world’s largest cohorts of mini-VSARR patients with a prolonged follow-up period including comprehensive echocardiographic evaluations. Despite the small sample size affecting the generalizability of our findings, we anticipate that ongoing enrollment in the MIRAGE multi-institutional registry will contribute to larger sample sizes in the future, allowing for more robust conclusions. Additionally, the surgical team’s experience with the mini-VSARR procedure and its potential influence on outcomes is not without meaning. Our team’s experience is derived from an extensive minimally invasive aortic valve replacement (AVR) program, where annual surgical volume plays a crucial role. We believe that gaining significant technical proficiency in mini-sternotomy AVR and right anterior mini-thoracotomy (RALT) AVRs serves as a foundation for undertaking mini-VSARR procedures. A gradual approach involving the incorporation of progressively complex surgical steps, such as supracoronary replacements with or without AVR, simple aortic valve repairs, Bentall procedures, and finally, David procedures, may further enhance the surgical team’s expertise in performing mini-VSARR effectively. On the other hand, there are inherent between-patient differences in the two reporting centers; firstly, the number of BAVs was significantly lower in the mini-VSARR than in the sternotomy-VSARR group; this may reflect the small sample size and early experience in BAV repairs in the mini-sternotomy reporting center; on the other hand, while full sternotomy BAV repairs already have long-term durability results [30,31], mini-access to root and BAV repairs are only limited to case studies and, therefore, highly anticipated. Secondly, BAV cases were also younger, which translated into age difference between the two cohorts. With BAV diagnosed, patients are followed closely and operated on much sooner than their tricuspid counterparts. Lastly, the size of the aneurysm was significantly smaller in the sternotomy-VSARR approach, but this reflects the two previous arguments. Thus, patients with diagnosed BAV undergo surgery sooner, that is, before severe AR and/or symptoms occur and before the size of the aneurysm reaches guidelines cut-off for surgery in tricuspid valve AR [6]. Altogether, it is also a reflection of extensive experience in aortic surgery in the sternotomy-VSARR center.

## 5. Conclusions

The presented study contributes to the evolving discourse on minimally invasive aortic surgery, particularly in the context of valve-sparing aortic root reimplantation. In conclusion, our findings, based on a limited patient cohort, suggest that the mini-VSARR approach exhibits non-inferiority in short-term safety and repair durability. However, the prolonged operative times necessitate further exploration in larger trials and meta-analyses. As technology and surgical expertise advance, the landscape of aortic surgery will continue to evolve, with minimally invasive approaches playing an increasingly prominent role in optimizing patient outcomes.

## Figures and Tables

**Table 1:** Preoperative characteristics.

Variable		All Patients			PS-Matched Patients		
		Sternotomy-VSARR (62)	Mini-VSARR (22)	P_value_	Sternotomy-VSARR (17)	Mini-VSARR (17)	P_value_
Age, years (median (IQR))		39 (28, 52)	64 (49, 65)	<0.001	63 (52, 68)	53 (48, 64)	0.143
Male gender		55 (88.7)	20 (90.9)	1.000	15 (88.2)	16 (94.1)	1.000
Diabetes		2 (3.2)	2 (9.1)	0.280	2 (11.8)	1 (5.9)	0.209
Smoking		26 (41.9)	9 (40.9)	1.000	8 (47.1)	7 (41.2)	1.000
Hypertension		39 (62.9)	18 (81.8)	0.119	16 (94.1)	14 (82.4)	0.601
CVD		2 (3.2)	1 (4.5)	1.000	1 (5.9)	0 (0.0)	1.000
Hyperlipidemia		16 (25.8)	7 (31.8)	0.589	5 (29.4)	3 (17.6)	0.280
BMI (median (IQR))		25.1 (23.3, 27.8)	27.8 (25.9, 30.7)	0.008	26.5 (24.6, 30.4)	27.1 (24.7, 29.3)	0.953
Pulmonary hypertension ^1^		3 (4.8)	0 (0.0)	0.563	0 (0.0)	0 (0.0)	NA
Renal impairment		10 (16.1)	6 (27.3)	0.343	7 (41.2)	5 (29.4)	0.721
NYHA							
	I	36 (58.1)	9 (40.9)	0.215	9 (52.9)	6 (35.3)	0.491
	II	21 (33.9)	8 (36.4)	1.000	6 (35.3)	6 (35.3)	1.000
	III	5 (8.1)	5 (22.7)	0.118	2 (11.8)	5 (29.4)	0.398
LVEF (%)(median (IQR)) ^1^		55 (51, 60)	59 (50, 60)	0.588	55 (53, 61)	59 (50, 60)	0.968
Previous MI		1 (1.6)	0 (0.0)	1.000	1 (5.9)	0 (0.0)	1.000
Previous PCI		0 (0.0)	1 (4.5)	0.262	0 (0.0)	1 (5.9)	0.354
Aortic disease							
	AV stenosis	2 (3.2)	0 (0.0)	1.000	1 (5.9)	0 (0.0)	0.354
	AV insufficiency	52 (83.9)	22 (100.0)	0.057	14 (82.4)	17 (100.0)	0.227
	Bicuspid aortic valve	27 (43.5)	1 (4.5)	0.001	3 (17.6)	1 (5.9)	0.601

^1^ missing data; VSARR, valve-sparing aortic root reimplantation; IQR, interquartile range; CVD, cerebrovascular disease; NYHA, New York Heart Association; LVEF, left ventricle ejection fraction; MI, myocardial infarction; PCI, percutaneous coronary intervention; AV, aortic valve.

**Table 2:** Surgical and in-hospital outcomes after propensity score-matching.

	Sternotomy-VSARR (17)	Mini-VSARR (17)	P_value_
CPB time (median (IQR))	166 (157, 177)	226 (220, 239)	<0.001
Aortic cross clamp (median (IQR))	140 (136, 147)	160 (158, 171)	<0.001
In-hospital mortality	0 (0.0)	0 (0.0)	NA
Cardiac tamponade and/or rethoracotomy for bleeding	3 (17.6)	2 (11.8)	1.000
Postoperative drainage (mL) (median (IQR))	845 (588, 1393)	740 (485, 1020)	0.651
Periprocedural MI	0 (0.0)	1 (5.9)	1.000
Respiratory failure	0 (0.0)	1 (5.9)	1.000
Neurologic complications	0 (0.0)	0 (0.0)	NA
Multiorgan failure	0 (0.0)	1 (5.9)	1.000
Acute kidney failure and/or dialysis	0 (0.0)	1 (5.9)	1.000
Sternal wound infection	0 (0.0)	1 (5.9)	1.000
ECMO	0 (0.0)	1 (5.9)	1.000
IABP	0 (0.0)	0 (0.0)	NA
HLoS (median (IQR))	9.50 (8.00, 11.00)	6.52 (4.92, 9.35)	0.031

VSARR, valve-sparing aortic root reimplantation; CPB, cardiopulmonary bypass; IQR, interquartile range; MI, myocardial infarction; ECMO, extracorporeal membrane oxygenation; IABP, intraaortic balloon pump; HLoS, hospital length of stay.

## Data Availability

Data are available upon request from the corresponding author.
